# The Polytechnic

**Published:** 1890-09

**Authors:** 


THE POLYTECHNIC. Chicago, Ill.: S. E. corner Madison Street and Fifth Avenue. Sample copy, ten cents.

This is the name of a new magazine to be published in Chicago, the initial number of which will be issued next month. Like the London magazine of that name, it will be the organ of a polytechnic institute, which, in this case, has been lately started in Chicago, and will be modeled after the famous London institute of similar name, an interesting account of which was given in the *Century* for June. The first number will be largely descriptive of the work of the institute, especially its trade schools, a peculiar feature of which is that students may earn their expenses while in attendance, and can learn almost any trade. As this promises to solve the vexed apprenticeship question, all master associations are warm supporters of the movement. An article on the new Evening Medical College, of Chicago, is also included in this number. The ladies will be interested in the description of the cooking, millinery, and dressmaking schools of the Chicago Polytechnic Institute.

